# Enhancing Sentinel Lymph Node Biopsy in Endometrial Cancer Using Augmented and Mixed Reality

**DOI:** 10.7759/cureus.77599

**Published:** 2025-01-17

**Authors:** Tsukuru Amano, Yutaka Yoneoka, Yuji Tanaka, Akimasa Takahashi, Shunichiro Tsuji

**Affiliations:** 1 Obstetrics and Gynecology, Shiga University of Medical Science, Shiga, JPN

**Keywords:** augmented reality, endometrial cancer, holography, robotic-assisted surgery, sentinel lymph node biopsy

## Abstract

Sentinel lymph node biopsy offers a less invasive alternative to full lymphadenectomy in endometrial cancer, reducing complications while maintaining diagnostic accuracy. This case report highlights the integration of holography and augmented reality (AR) in a 34-year-old woman undergoing robotic-assisted surgery for endometrial cancer. Preoperative imaging combined with single-photon emission computed tomography and computed tomography identified sentinel lymph nodes, which were visualized using mixed reality (MR) technology. This approach enabled the surgical team to accurately understand the three-dimensional spatial relationships between lymph nodes and surrounding critical structures. Holographic projections provided precise guidance during surgery, improving lymph node identification and minimizing invasiveness. No lymph node metastasis was detected, but a diagnosis of the International Federation of Gynecology and Obstetrics (FIGO) stage IIIA was confirmed due to tumor seeding in the peritoneum. The patient underwent successful adjuvant chemotherapy with no recurrence observed. This report demonstrates the significant potential of holography and AR to enhance spatial awareness and surgical precision. These technologies represent a promising advancement in sentinel lymph node biopsy for patients with gynecologic cancers, contributing to reduced surgical invasiveness and alleviating stress for surgeons.

## Introduction

Sentinel lymph node (SLN) biopsy in endometrial cancer offers a less invasive alternative to full lymphadenectomy, reducing complications such as lymphedema while maintaining high diagnostic accuracy for lymph node metastasis detection [1]. SLN detection typically involves the combined use of the radioisotope (RI) method with 99m technetium (99mTc)-labeled phytate and the near-infrared fluorescence imaging method with indocyanine green (ICG) [2-4]. While the RI method is effective for detecting SLNs, it does not provide visual feedback during surgery, necessitating the use of ICG as an adjunct. However, the ICG method also faces challenges that remain, such as the identification of multiple SLNs, including secondary SLNs, and the difficulty of locating SLNs in cases such as obese patients [4]. Therefore, a technology that clearly visualizes the SLNs identified by single-photon emission computed tomography combined with computed tomography (SPECT/CT) is desired. Recently, technologies such as augmented reality (AR) and mixed reality (MR) have found applications in the field of surgery [5-10]. MR allows users to experience both the physical world and virtual elements simultaneously by accurately aligning images within spatial coordinates. By integrating MR into surgical procedures, particularly through the use of head-mounted displays (HMDs), surgeons can access advanced visualization tools to view three-dimensional holographic models tailored to individual patients during operations. This case report examines the application of holography-guided SLN biopsy using reality to visualize RI-identified SLNs in three-dimensional (3D) space during surgery.

## Case presentation

**Figure 1:**
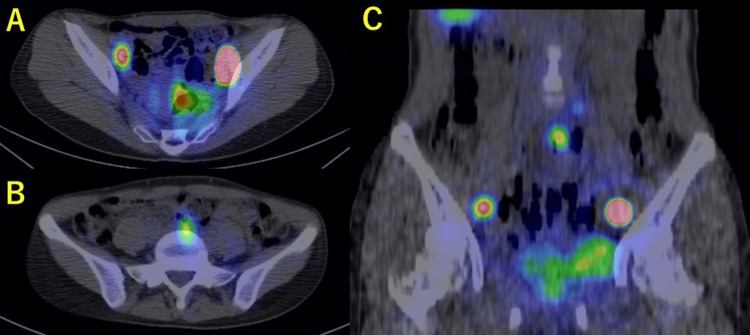
Sentinel lymph node identified by SPECT/CT. (A and C) Bilateral obturator lymph node; (B and C) presacral lymph node. SPECT/CT, single-photon emission computed tomography combined with computed tomography

**Figure 2:**
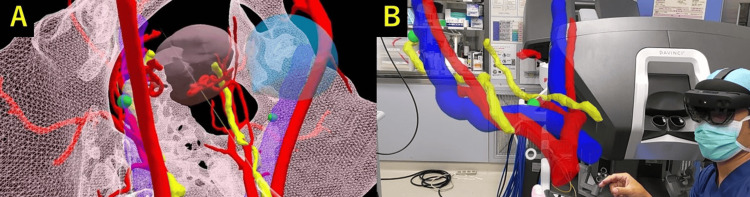
Holograms in the pelvis. (A and B) Holograms showing the positional relationship between blood vessels, ureters, and sentinel lymph nodes. The green spheres represent the sentinel lymph nodes.

**Figure 3:**
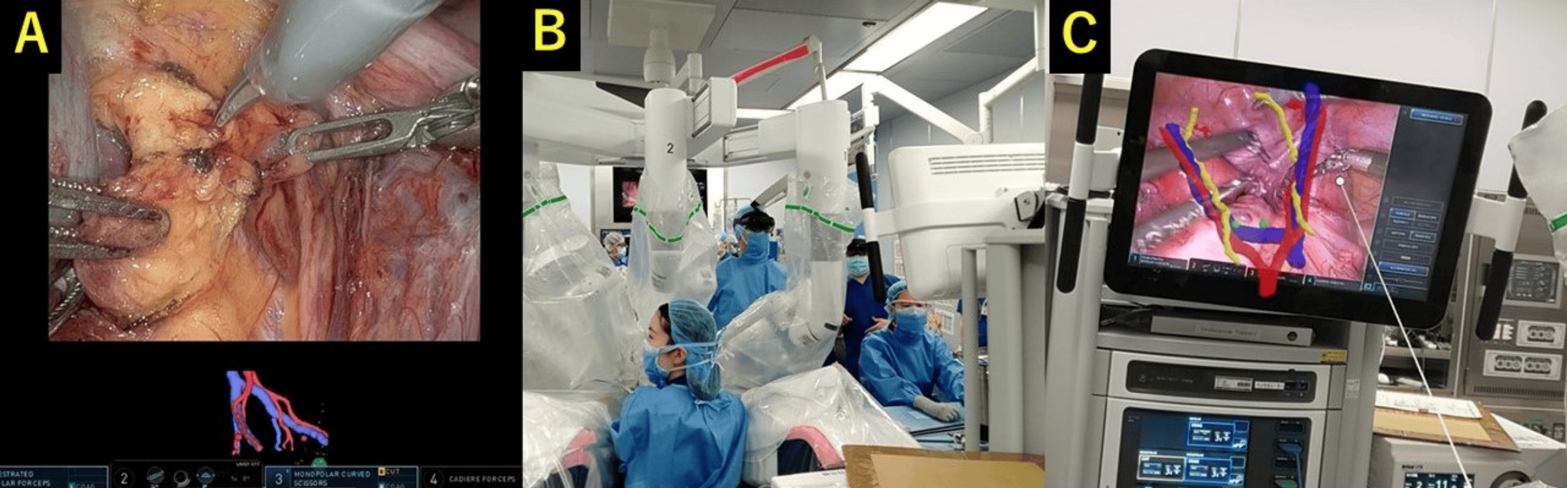
Robot-assisted laparoscopic sentinel lymph node biopsy under hologram guidance. (A) Displaying the 3D image within the viewer of the surgeon console using the da Vinci TilePro (Intuitive Surgical, Sunnyvale, CA) function. (B) A bedside assistant wearing HoloLens2 (Microsoft Corp., Redmond, WA). (C) With the see-through function, the assistant can overlay the surgical monitor with the 3D hologram for simultaneous viewing.

A 34-year-old woman, gravida 0 para 0, presented with abnormal genital bleeding. Transvaginal ultrasound and magnetic resonance imaging (MRI) revealed a 2.5 cm mass confined to the uterine isthmus. Histological examination indicated endometrioid carcinoma, leading to a diagnosis of early-stage endometrial cancer. Contrast-enhanced CT showed no evidence of extra-uterine lesions. A robot-assisted laparoscopic hysterectomy, bilateral salpingo-oophorectomy, and SLN biopsy were planned. SLN detection was performed using a cervical injection of 99mTc-labeled phytate radioisotope. SPECT/CT identified SLNs in the right obturator lymph node, left obturator lymph node, and presacral lymph node (Figures 1A-1C). Preoperative 3D CT images were generated, and stereolithography (STL) data were created using Vincent software (Fujifilm Corp., Tokyo, Japan). The STL data were then imported into AR software *Holoeyes* (Holoeyes Inc., Tokyo, Japan) and displayed on a head-mounted display (HMD) using HoloLens2 (Microsoft Corp., Redmond, WA; Figures 2A, 2B). Preoperatively, the surgical team utilized HoloLens2 to visualize the SLNs and their anatomical relationships to surrounding structures such as blood vessels and the ureters. During surgery, these 3D images were displayed on operating room monitors, the da Vinci vision cart, and in a virtual space via HoloLens2 worn by a bedside assistant (Figures 3A-3C). This allowed the team to better understand the spatial relationships between the SLNs, blood vessels, and ureters, improving the precision of SLN biopsy.　The right obturator lymph node, left obturator lymph node, left internal iliac lymph node, and presacral lymph node were biopsied and removed using a sleeve device for external extraction. Radiation measurements confirmed that the nodes were SLNs. Rapid pathological examination of the nodes revealed no metastasis, so full lymphadenectomy was omitted. Additionally, a small nodule (<1 cm) was observed in the peritoneum of the pouch of Douglas, and a biopsy of this nodule was also performed. The total operative time was 3 hours and 40 minutes, and the console time was 2 hours and 45 minutes. Total blood loss was minimal. There were no intraoperative or postoperative complications. The histopathological diagnosis confirmed endometrioid carcinoma, grade 1, with 6 mm/20 mm myometrial invasion and no lymphovascular space invasion. The bilateral adnexa was free of metastasis. However, the biopsy from the peritoneum revealed a small focus on tumor seeding, leading to a diagnosis of the International Federation of Gynecology and Obstetrics (FIGO) stage IIIA. The patient subsequently received six courses of adjuvant chemotherapy with carboplatin and paclitaxel. At the time of writing, there has been no evidence of disease recurrence.

## Discussion

Two major clinical issues have been identified. In SLN biopsy for pelvic lymph nodes in endometrial cancer, holography-guided MR technology enhances the surgeon's spatial awareness and facilitates the procedure. The assistant can more easily identify the target lymph node designated by the surgeon, enabling more precise and appropriate maneuvers.

First, in the biopsy of SLNs for pelvic lymph nodes, MR technology guided by holography improves the surgeon's spatial perception and streamlines the surgical process. There have been several reports on surgical support using holography-guided MR technology to date. A report on holographic image-guided thoracoscopic surgery for patients with esophageal cancer indicated that the use of holography for surgical assistance contributed to improved safety and precision, especially in patients with abnormal blood vessels [6]. In reports on intraoperative holography support using MR technology in liver surgery, it is expected to be effective in enhancing spatial awareness, facilitating information sharing among surgeons, and providing intuitive operability. It is considered particularly useful as a preoperative simulation just before surgery rather than for intraoperative navigation [7]. In laparoscopic cholecystectomy, the holography navigation system using MR technology has been reported to be highly effective in enhancing spatial awareness and deepening surgical understanding, particularly for less experienced surgeons [8]. Additionally, the usefulness of MR technology has also been reported in surgeries for rectal cancer and retroperitoneal tumor resections [9,10]. These reports indicate that surgical support using holography-guided MR technology is useful for the resection of fixed targets. Lymph nodes and blood vessels are also fixed structures that do not move even when the body position or orientation changes, making holography-guided AR technology highly compatible with such procedures. In this case, a hologram was created by superimposing the 3D reconstruction of arteries, veins, and ureters based on contrast-enhanced CT scans with the center of the SLN detected by SPECT-CT, using the pelvic bone as a reference. As a result, there was complete concordance between the SLN location visualized through the holography and its actual anatomical position. Holography-guided AR technology enables precise measurements, such as determining how many centimeters caudal the target lymph node is from the bifurcation of the internal and external iliac arteries, significantly simplifying lymph node identification. Furthermore, in SLN biopsy, only the targeted lymph nodes are removed while preserving other lymph nodes as much as possible, which further enhances the utility of this technology.

Second, the assistant can more readily recognize the lymph node targeted by the surgeon, allowing for more accurate and effective actions. In robotic surgery, an assistant wearing a see-through HoloLens can overlay the endoscopic camera view with holographic images, allowing them to understand the lymph nodes targeted by the surgeon and provide appropriate assistance effectively. This improves surgical accuracy, facilitates smoother coordination between the surgeon and the assistant, and is expected to reduce the risk of complications and shorten the operation time.

The RI method is one of the excellent techniques for identifying SLNs, but it has the drawback that the surgeon cannot visually confirm the target lymph nodes during the operation. The ICG method is one approach to compensate for this drawback; however, it also has issues such as identifying multiple lymph nodes, including secondary SLNs, and difficulties in visualization in cases like obesity [4,11]. Holography-guided SLN biopsy using AR technology offers enhanced intraoperative spatial awareness, particularly in cases where the identification of SLNs is challenging due to anatomical complexity or patient obesity. By visualizing SLNs in 3D space and improving the understanding of their relationships with critical structures such as blood vessels and ureters, this approach facilitates more accurate and less invasive biopsies. At present, the number of cases is limited, and challenges remain in generalizing this technology. Although larger studies are needed to further validate the utility of AR in SLN biopsy for gynecologic cancers, this technology shows promise in improving surgical outcomes and reducing morbidity for patients with endometrial cancer.

## Conclusions

Holography-assisted SLN biopsy is a valuable tool in the surgical management of endometrial cancer. Enhancing the surgeon’s spatial awareness allows for more accurate SLN identification and biopsy, particularly in anatomically challenging cases. This case demonstrates the successful use of AR to guide SLN biopsy, resulting in an effective and minimally invasive surgical procedure.
